# Applications and analysis of targeted genomic sequencing in cancer studies

**DOI:** 10.1016/j.csbj.2019.10.004

**Published:** 2019-11-07

**Authors:** Findlay Bewicke-Copley, Emil Arjun Kumar, Giuseppe Palladino, Koorosh Korfi, Jun Wang

**Affiliations:** a Centre for Cancer Genomics and Computational Biology, Barts Cancer Institute, Queen Mary University of London, Charterhouse Square, London EC1M 6BQ, UK; b Centre for Haemato-Oncology, Barts Cancer Institute, Queen Mary University of London, Charterhouse Square, London EC1M 6BQ, UK

**Keywords:** Targeted sequencing, Variant calling, PCR duplicates, Background error, Cancer genomics, Clinical samples, BWA, Burrows-Wheeler Aligner, FF, Fresh Frozen, FFPE, Formalin Fixed Paraffin Embedded, CLL, Chronic Lymphocytic Leukaemia, FL, Follicular Lymphoma, tFL, Transformed Follicular Lymphoma, NSCLC, Non-Small Cell Lung Carcinoma, NHL, Non-Hodgkin Lymphoma, GATK, Genome Analysis Toolkit, COSMIC, Catalogue of Somatic Mutations in Cancer, ESP, Exome Sequencing Project, ICGC, International Cancer Genome Consortium, NCCN, the National Comprehensive Cancer Network®, TCGA, The Cancer Genome Atlas, QC, Quality Control, BAM, Binary Alignment Map, SAM, Sequence Alignment Map, VAF, Variant Allele Frequency, NGS, Next Generation Sequencing, WES, Whole Exome Sequencing, WGS, Whole Genome Sequencing, TS, Targeted Sequencing, UMI, Unique Molecular Identifiers, MBC, Molecular Barcode

## Abstract

Next Generation Sequencing (NGS) has dramatically improved the flexibility and outcomes of cancer research and clinical trials, providing highly sensitive and accurate high-throughput platforms for large-scale genomic testing. In contrast to whole-genome (WGS) or whole-exome sequencing (WES), targeted genomic sequencing (TS) focuses on a panel of genes or targets known to have strong associations with pathogenesis of disease and/or clinical relevance, offering greater sequencing depth with reduced costs and data burden. This allows targeted sequencing to identify low frequency variants in targeted regions with high confidence, thus suitable for profiling low-quality and fragmented clinical DNA samples. As a result, TS has been widely used in clinical research and trials for patient stratification and the development of targeted therapeutics. However, its transition to routine clinical use has been slow. Many technical and analytical obstacles still remain and need to be discussed and addressed before large-scale and cross-centre implementation. Gold-standard and state-of-the-art procedures and pipelines are urgently needed to accelerate this transition. In this review we first present how TS is conducted in cancer research, including various target enrichment platforms, the construction of target panels, and selected research and clinical studies utilising TS to profile clinical samples. We then present a generalised analytical workflow for TS data discussing important parameters and filters in detail, aiming to provide the best practices of TS usage and analyses.

## 1 Introduction

Recent advances in next-generation sequencing technology have revolutionised our understanding of cancer biology and clinical research. It is now more affordable than before to carry out large-scale NGS experiments with a reasonable turnaround time. This has led to a rapidly expanding body of pioneering research exploring the genomic landscape and molecular mechanisms of various cancer types, as well as the discovery of genetic drivers (i.e., mutations that confer a selective growth advantage, thus promoting cancer development), exemplified by the effort from large international sequencing initiatives, such as The Cancer Genome Atlas (TCGA) and the International Cancer Genome Consortium (ICGC). This has generated vast amounts of data and identified numerous biomarkers and targets for patient stratification and therapeutics. Although the translation of these findings into the clinic has been slow, in certain settings, NGS is becoming a complementary diagnostic tool, guiding the decision making to achieve personalised and/or precision medicine in a number of cancers [1], [2], [3], [4], [5], [6]. With the magnitude of sequencing data generated, the continuing development of advanced bioinformatics tools capable of handling these data efficiently in a timely manner is vital for NGS-centred research and clinical implementation. Researchers and clinicians are now faced with a wide range of NGS techniques and platforms with no clear consensus guidelines, where the trade-offs between costs, accuracy, power and technical difficulties must be considered.

**Fig. 1:**
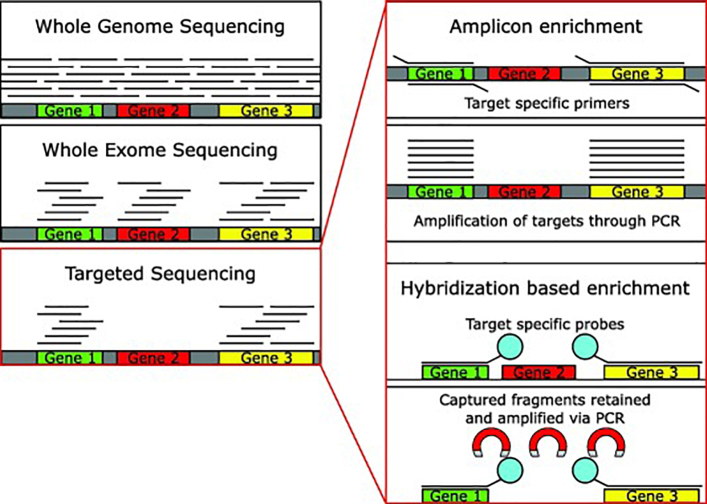
Methods of DNA-seq. Whole genome sequencing, whole exome sequencing and targeted sequencing are illustrated. For the latter, the two library preparation approaches are shown.

There are three main types of NGS sequencing of DNA that can be used for the identification of genomic mutations: whole-genome sequencing, whole-exome sequencing and targeted sequencing (Fig. 1). We summarise and compare the key information of these three platforms in Table 1. Compared to WGS and WES, TS, is a powerful approach that can fulfil the best balance between the accurate identification of targeted events with great sensitivity, and the overall cost and data burden for large-scale executions. For the data analysis, many existing methods and pipelines designed for WGS/WES can be applied to TS. However, due to the high depth of TS, extra care needs to be taken during the analysis to ensure only high-quality variant calls are retained, especially for data generated from low quality or fragmented DNA and/or without matched normal control. Currently, as with other types of NGS, there is still a lack of gold-standard pipelines for TS analysis, which can lead to poor reproducibility between laboratories, even for the same data sets. This greatly affects the accuracy and efficacy of TS in calling variants for prognostic and therapeutic signatures, as different labs working with different pipelines may not reliably call these variants.

**Table 1:** Different types of Next Generation Sequencing for genomics.

Platform	Cost (per sample, USD)	Sites	Region Size (bp)	Depth	Data size (Processed Bam)
WGS	$1000–$3000	All coding and non-coding regions	~3 × 10^9^	30–60×	Depending on coverage ~60 GB–350 GB
WES	$500–$2000	Exonic regions	~6 × 10^7^	150–200×	Depending on coverage ~5 GB–20 GB
TS	$300–$1000	Specifically targeted regions	Varies by panel size ~1 × 10^5^–1 × 10^7^	200–1000×+	Varies by panel size and coverage ~100 MB–5 GB

Bearing all these in mind, in this review we first present a general overview of TS, associated platforms and their implementations in various cancer studies. We show that TS provides a powerful and versatile tool to profile clinical samples in cancer research and clinical trials. We then present a generalised analytical workflow for TS data, with commonly used software, and important parameters and filters discussed in each step. We aim to provide guidance on how to analyse the data in a more standardised manner.

## 2 Targeted genomic sequencing

TS focuses on a number of targeted regions often including many known drivers or clinically-actionable genes of interest and identifies sequence variants with high confidence and accuracy. For example, the genes *KRAS* and *TP53* are often targeted across a range of cancer types, as they are commonly found to be mutated with a number of hotspots. *BRAF* and *EGFR* are also screened in many solid tumours, as they contain clinically relevant mutations [7], [8], [9], [10], [11], [12], [13].

The great sequencing depth utilised in TS (e.g., ultra-deep sequencing at a depth of 10,000x and higher) makes it very powerful for profiling clinical samples, such as formalin fixed paraffin embedded (FFPE) and circulating tumour DNA (ctDNA) where DNA quality and/or tumour content is low. Greater depth of coverage also allows TS to pick out mutations that are present only in a small fraction of malignant cells (i.e., sub-clonal), and in the setting of detecting minimal residual disease, with variant allele frequency (VAF) sufficiently detected as low as 0.1–0.2% [14], [15], [16]. All these attributes ensure that TS is superior to non-NGS based techniques (e.g., Sanger sequencing and digital PCR) and WGS/WES in large-scale genomic testing and clinical trial setting (see comparison details in Table 2).

**Table 2:** A comparison of targeted methods of genomic analysis.

Platform	Target size	Cost (per sample, USD)	Massively Parallel	Minimum allele frequency	Purpose in Research
TS	~1 × 10^5^–1 × 10^7^ bp	$300–$1000	True	1% (without error suppression)	Discovery/Validation
Sanger Sequencing	300–1000 bp	<$30	False	~15%	Validation
Digital PCR	1–80 bp	<$10	False	<0.001%	Validation

TS has been widely used in cancer studies and clinical trials to stratify patients into risk groups based on the mutational status of key genes [17], [18], [19], [20], [21], [22], [23]. In clinical practice, Foundation Medicine has launched the first FDA-approved broad companion diagnostic (CDx) that is clinically and analytically validated for solid tumours. This platform identifies genomic alterations and biomarkers across 300+ genes with a median depth of coverage of 500x. It is suitable for processing FFPE samples with quick turnaround (<2 weeks), offering invaluable information for therapeutic targets and immunotherapy biomarkers.

### 2.1 Methods of targeted sequencing

Targeted sequencing comes in two main forms, amplicon or capture-based (Fig. 1). Amplicon-based enrichment utilises specifically designed primers to amplify only the regions of interest prior to library preparation [24]. Alternatively, in capture-based approaches, the DNA is fragmented and targeted regions are enriched via hybridization oligonucleotide bait sequences attached to biotinylated probes, allowing for isolation from the remaining genetic material [24], [25]. Amplicon-based enrichment is the cheaper of the two technologies and shows a greater number of on target reads; however, the coverage of these regions is more uniform with hybrid sequencing [24], [26]. Some commercially available amplicon platforms attempt to address the coverage issues by using specific primers that are able to amplify overlapping fragments in a single PCR reaction [27]. Amplicon based sequencing requires much less starting material than hybrid-capture, making it ideal if there is little DNA available for TS.

Hybrid-capture has been shown to produce fewer PCR duplicates than amplicon enrichment (<40% and up to ~80%, respectively) [24]. These duplicates are also more trivial to remove computationally, as the random shearing of the DNA in hybrid-capture platforms reduces the likelihood of two unique fragments aligning to the same genomic coordinates compared with the identical amplicons generated by amplicon enrichment platforms. This makes hybrid-capture especially useful for samples where these PCR artefacts are more likely to occur, such as FFPE and ctDNA samples. Further, certain regions of the genome make primer design for amplicon enrichment difficult (e.g. regions with a high number of repeated sequences). The long bait sequences used in hybrid-capture, however, allow a greater level of specificity in region selection. Overall hybrid-capture based platforms provide more accurate and uniform target selection, whilst amplicon-based platforms are often used in small scale experiments where sample quantity or cost are a factor.

### 2.2 Platforms for targeted sequencing

There are several commercially available platforms for these two approaches. Many of these platforms are also used for WES. An outline of these platforms is shown in Table 3. Despite the differences between the various platforms, they have been shown to lead to relatively concordant variant calling [24].

**Table 3:** An overview of some commercially available TS platforms.

Platform	Company	Enrichment	Protocol overview
Ion AmpliSeq™	Thermo Fisher Scientific	Amplicon	Targeted regions are amplified through target specific primers. These primers are removed, the sequencing adapters are added and the amplicons are amplified again to generate the library. Needs to be sequenced using Ion Torrent™ Sequencer.
Access Array	Fluidigm	Amplicon	Amplifies target regions, adding an overhanging universal adapter. The universal adapter is then bound by the sequencing adapters. Can be sequenced on both Ion Torrent™ and Illumina platforms.
Haloplex^HS^	Agilent	Amplicon	Circularises restriction enzyme fragmented gDNA using biotinylated probes. Probes captured using magnetic streptavidin beads. Circular molecules are then amplified to generate a linear library.
GeneRead DNAseq Targeted Panels V2	Qiagen	Amplicon	Targeted regions amplified via multiplexed PCR-based enrichment. The samples are pooled, and the amplicons are purified using AMPure XP beads. Sequencing library can then be created using a platform specific kit.
TruSeq Amplicon	Illumina	Amplicon	Probes are bound at either end of a targeted region. The region is amplified via PCR, leaving an amplicon of the region with probes either end. Indices and sequencing adapters are then bound to the overhanging ends of the probes.
SureSelect^XT^	Agilent	Hybridization	Fragmented gDNA is amplified and the targeted regions are captured using target specific biotinylated probes. These probe-bound fragments are isolated and amplified to create the library.
SeqCap EZ	Roche Nimblegen	Hybridization	Fragmented gDNA is amplified, the sequencing adapters are added, and these fragments are then amplified. Target specific probes are added and probe bound fragments are isolated to generate the library.
Cell3™Target	Nonacus	Hybridization	DNA is enzymatically fragmented and Illumina Unique Molecular Identifier (UMI) containing adapters are ligated. The fragments are amplified prior to target enrichment using biotin-labelled probes and streptavidin coated beads. The enriched fragments are amplified again and then sequenced on an Illumina sequencer.

### 2.3 Use and design of panels for targeted sequencing

#### 2.3.1 Targeted panel construction

The term targeted panel is used here to refer to the collection of genomic coordinates that are of interest to the user. An important difference between WES panels and targeted panels, is that TS is not constrained to canonical gene targets and can target other regions, such as promoters [28] or breakpoints [29].

There are commercially available targeted gene panels, usually designed for research [30], [31] or clinical purposes [32], [33]. They are designed to amplify genomic regions that are known to be of interest within cancer, or specific cancer subtypes. Using these panels greatly speeds up the process of the sequencing as they have already been designed, tested and validated.

Commonly, however, users design their own customised panels dependent on their research questions, although thorough target validation of these panels is needed before use. Customised panels are often generated by a thorough review of the current literature and cross referencing publicly available cancer mutation resources such as TCGA, ICGC, CbioPortal, and Catalogue of Somatic Mutations in Cancer (COSMIC) (http://cancer.sanger.ac.uk) databases [34], [35], [36], [37], [38], selecting genes that are frequently mutated, and targets that have been functionally validated in that cancer. In many cancer studies, an initial discovery cohort has been initially profiled with WGS or WES to the identify significantly mutated genes (via algorithms like MutSigCV [39], dNdScv [40], oncodriveFM [41]). These genes are then selected for TS with higher depth in the validation cohort(s) to establish their validity and frequencies [42], [43], [44], [45]. Examples of the applications of these panels are included in the next section.

#### 2.3.2 Applications of targeted gene panels in cancer studies

There are a large body of clinical studies that utilise genomic TS for research on clinical samples. Some recent examples have been listed in Table 4 [17], [43], [44], [45], [46], [47], [48], [49], [50], with targeted panels ranging from as few as 25 genes [44] to 122 genes [49]. These studies illustrate that a wide range of TS platforms, sequencing depths, data processing and variant calling methods were used.

**Table 4:** Selected example of studies that analysed mutations using targeted DNA sequencing in human samples.

Disease	Tissue Origin	Authors	Journal	Genes	Depth	Platform	Target capture mode	Machine	FFPE/fresh frozen (FF)	Duplicate handling	Variant Calling
Acute Myeloid Leukaemia	Tumour	Ivey et al. 2016	New England Journal of Medicine	51	1280x	Agilent Haloplex^HS^	Amplification	HiSeq 2000	Not Reported	Not Reported	VarScan2
	Normal Peripheral blood	Abelson et al. 2018	Nature	111	Not Reported	Roche NimbleGen	Hybrid Capture	HiSeq 2000	FF	MBC	Varscan2
						Agilent SureSelect					Shearwater ML Pindel
Breast Cancer	Tumour	Ellis et al. 2012	Nature	Variable	Not Reported	Roche NimbleGen	Hybrid Capture	Not Reported	FFPE	Picard	VarScan2
											BreakDancer
	Germline	Couch et al. 2015	Journal of Clinical Oncology	122	300x	Illumina TruSeq Amplicon	Amplification	HiSeq™ 2000	Not Reported	Not Reported	GATK Unified Genotyper/SAMtools
FL	Tumour	Okosun et al. 2014	Nature Genetics	28	840x	Fluidigm Access Array™	Amplification	Miseq	FF	Not Reported	VarScan2
	Tumour	Pastore et al. 2015	The Lancet Oncology	74	Not Reported	Agilent SureSelect	Hybrid Capture	HiSeq 2500	FFPE	Picard	MuTect
											Indel Locator
	Tumour	Araf et al. 2018	Leukaemia	25	8000x	Agilent Haloplex^HS^	Amplification	MiSeq	FFPE	UMI	VarScan2
Pancreas	Tumour	Sausen et al. 2015	Nature Communications	116	754x	Agilent SureSelect	Hybrid Capture	HiSeq 2000/25000 & MiSeq	Both	CASAVA	VariantDx
Skin Cancer	Normal Skin	Martincorena et al. 2015	Science	74	500x	Agilent SureDesign	Hybrid Capture	HiSeq 2000/25000	FF	Picard	Shearwater ML

## 3 Guidance for analysis of targeted genomic sequencing

**Fig. 2:**
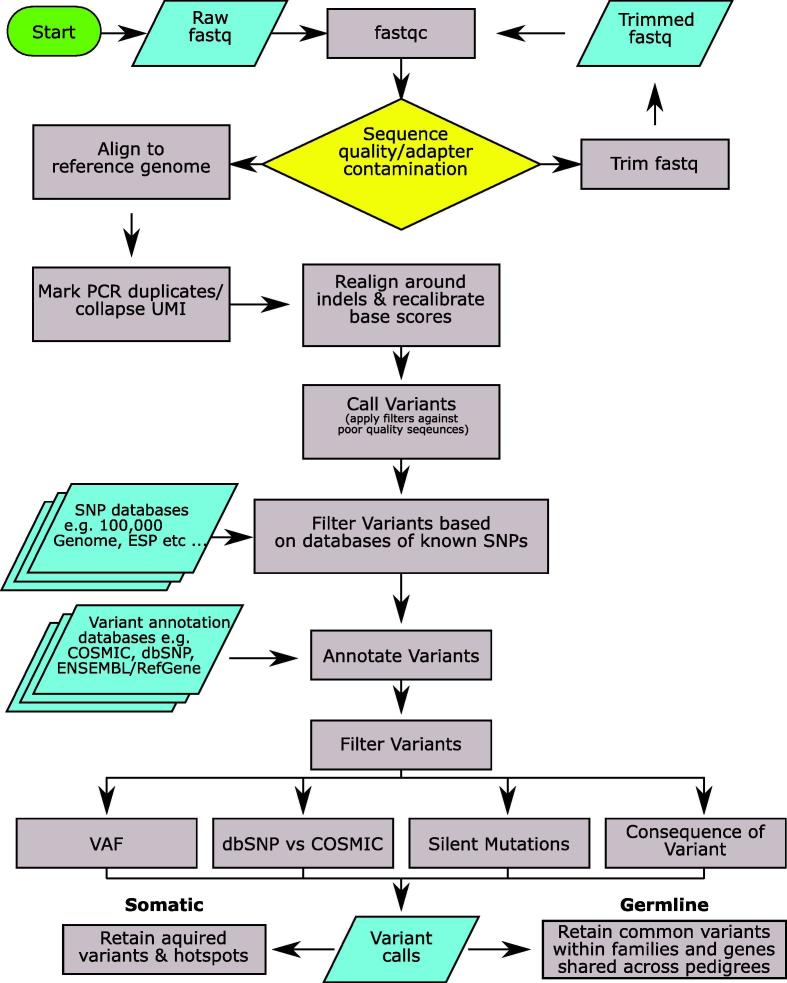
A generalised workflow for calling variants in clinical samples. This workflow includes quality check, sequence alignment and further processing, variant calling, annotation and filtering.

In this section we provide detailed guidance for the analysis of TS, from initial quality control (QC) and data pre-processing, to variant calling, annotation and filtering (Fig. 2). Commonly used methods and software in each step and important parameters/filters are discussed, aiming to provide readers a comprehensive overview of the whole analytical process from raw reads to high-confidence annotated calls. We further focus on PCR duplication marking/removal and variant filtering in greater depth, as these are crucial steps to ensure the best quality variant calls. Key steps of TS data analysis and commonly used software are listed in Table 5.

**Table 5:** Steps and commonly used software for the processing of targeted sequencing data.

Step	Software
QC	FastQC, CutAdapt [51], bedtools [52]
Alignment	BWA [53], Bowtie2 [54], Torrent Suite
PCR Duplicates Handling or Unique Molecular Identifier /Molecular Barcode (MBC) deconvolution	Duplicates - Picard, SAMtools [55], Torrent Suite UMI/MBC – fgbio, Agilent Genomics NextGen Toolkit, Gencore, Connor
Realignment and base score recalibration	Genome Analysis Tool Kit (GATK) [56]
Variant calling	MuTect2 (GATK), Strelka2 [57], VarScan2 [58], HaplotypeCaller (GATK), Torrent Suite, Pindel [59], Ion Reporter Software, VariantDX
Annotation	Annovar [60], snpEFF [61], Variant Effect Predictor [62]

### 3.1 Quality control and data pre-processing

#### 3.1.1 QC and alignment

The first step of all NGS pipelines is to assess the quality of the sequenced reads, using FastQC (http://www.bioinformatics.bbsrc.ac.uk/projects/fastqc). It summarises and visualises base quality score for every base pair sequenced, which allows users to have an overview of the read quality and decide whether a trimming step is needed, especially at the 3′ end where the base quality is often lower. FastQC also produces summarised information of adapter fragment contamination and GC content within all reads. This analysis determines whether adapter fragments have been incorporated into the reads and need to removed using software such as CutAdapt [51]. The GC content of the reads is useful to indicate whether the sample is contaminated with DNA from another organism, as this would likely lead to a secondary peak due to the different GC content of that genome [63].

Next, raw or trimmed reads are aligned to the reference genome to generate Sequence Alignment Map (SAM) or Binary Alignment Map (BAM) files for each sample. Commonly used aligners include the Burrows-Wheeler Aligner (BWA) [53] and Bowtie2 [54]. Ion Torrent™ also have their own customised aligner specifically for working on data generated from their platform. Within alignment files the mapping quality score (i.e., the likelihood of a read mapping to multiple locations in the genome) is recorded for each read, in addition to their mapped coordinates.

It should be noted that the experimental and web-lab quality of TS experiments is also a key determinant of the sequencing data quality, such as how fragmented the DNA is, and the amount of input DNA. Low quantity of input DNA will require more PCR cycles, leading to a high level of PCR duplicates and limiting the achievable depth of coverage of the experiment. Monitoring the experimental quality of TS is always part of good laboratory practice, ensuring the highest quality of sequencing data in the downstream analyses. It is also important to check for germline/tumour mix-ups and contamination whilst running the pipeline. Whilst these errors are very difficult to determine from the FASTQ files alone, they may become more apparent in the later analytic stages, such as variant calling and VAFs, e.g. a large number of variants called in the germline that are absent in the tumour sample.

#### 3.1.2 Assessment of off-target reads

Various QC steps should always take place to ensure the best quality of TS data. As TS focuses on regions of interest in the design panel, we expect the majority of reads generated should come from targeted regions, however, off-target reads are a common occurrence. After alignment, the percentage of reads that cover targeted regions can be assessed using software such as bedtools [52], and the GATK coverage module. A high proportion of off-target reads may indicate that the TS experiment has failed, or the targeted regions contain too many repeat sequences. This could be possibly adjusted by making the capture or library preparation process more efficient, e.g., adjust input DNA to beads ratio, and wash more stringently. With a large panel of hundreds of targeted genes, roughly >70% of the reads aligning to the targeted regions is a positive indicator of a good quality TS data set [26].

#### 3.1.3 Marking and removal of PCR duplicates

PCR duplicates are sequence reads that align to the same genomic coordinates and typically arise during PCR steps in the library preparation. The duplication rate tends to be much higher for fragmented DNA of low quality, e.g. FFPE and ctDNA, reaching ~50–60% for some cases, while for FF DNA, this rate is usually less than 20%. These PCR duplicates need to be marked and removed before any downstream analysis, as including them will lead to overestimation of coverage in targeted regions, and more importantly result in incorrect allele frequency estimation.

A number of software are used to search for PCR duplicates within aligned NGS data. A commonly used program is the MarkDuplicates function within Picard Tools (http://broadinstitute.github.io/picard/). This tool looks for reads with the same start and end coordinates and then add tags to the bam files that mark these reads as duplicates. Another tool, SAMtools rmdup, simply outright removes the duplicate reads retaining the read with the highest mapping quality [55]. However, these software based attempts cannot discriminate between two unique reads that happen to align in the same position by chance and actual duplicates [64]. There are additional molecular techniques, such as Unique Molecular Identifiers or Molecular Barcodes (MBC), available to ensure only unique reads are measured in the downstream analysis. These are exemplified by the Nonacus Cell3™ Target, Agilent Haloplex^HS^ and SureSelect^XT^ platforms.

**Fig. 3:**
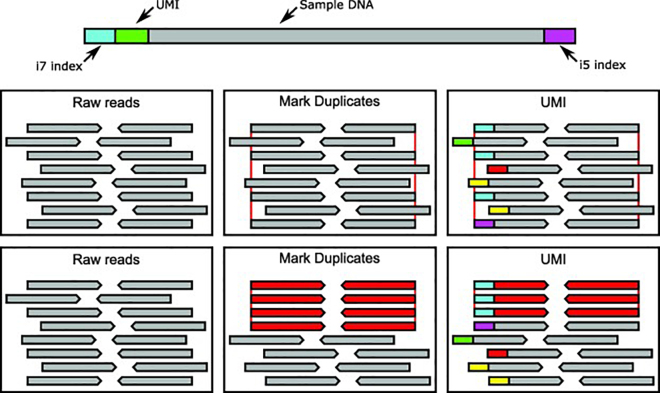
Considering duplicates in next generation sequencing. PCR duplicates can occur during the course of NGS. Whilst duplicates will appear to be separate reads, they are actually technical noise due to errors during PCR and sequencing. The two methods of correcting these errors are detailed above. The red lines indicate reads the start and end coordinates of the duplicates. Reads are coloured based on whether they are considered individual reads (grey) or duplicates (red), the coloured bars at the start of each read in the UMI panel represent different UMI sequences. In the above situation marking duplicates would cause 4 reads to be combined into a single read whereas the UMI based duplicate method is able to distinguish between true duplicates and unrelated reads with the same coordinates. (For interpretation of the references to colour in this figure legend, the reader is referred to the web version of this article.)

UMIs or MBCs are random short nucleotide sequences that are ligated to the DNA fragments during the library preparation. These sequences act as barcodes that mark each read as coming from the amplification of a single fragment, providing a more accurate mechanism for determining PCR duplicates. The different methods of PCR duplicate handling are outlined in Fig. 3. An outline of the additional steps required for UMI/MBC workflow are as follows:

• Align the reads to the reference genome first as usual, with all the barcodes contained in a separate file.
• The reads are then grouped by their barcodes to ensure the duplicate reads are found next to one another.
• The reads with the same UMIs are then collapsed to create consensus reads, with all duplicated reads removed. Available programs to deal with UMIs or MBCs include ‘fgbio’ (http://fulcrumgenomics.github.io/fgbio), The Agilent Genomics NextGen Toolkit (AGeNT) (Agilent Technologies, http://www.genomics.agilent.com), Gencore (https://github.com/OpenGene/gencore) and Connor (https://github.com/umich-brcf-bioinf/Connor).

**Fig. 4:**
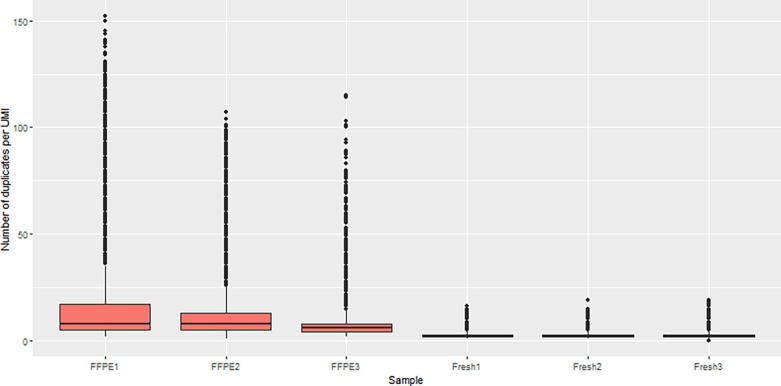
The number of UMIs found in common across FFPE and FF clinical samples. FF and FFPE follicular lymphoma biopsies were sequenced using the Nonacus hybrid capture platform (unpublished in-house data for demonstration purposes). The number of UMI tagged duplicates that were found in these samples were counted. Only consensus reads with at least two duplicate reads were considered in this analysis.

Fig. 4 demonstrates the effect of UMI duplicates from FFPE and FF samples from follicular lymphoma biopsies (unpublished in-house data, used here for demonstration purpose only). These samples were processed using a custom hybrid capture panel from Nonacus run in-house at BCI. The number of duplicate reads found to share the same UMI were counted for each sample. While FF samples had an average of 2–3 duplicated reads per consensus sequence, FFPE samples had a far greater number of PCR duplicates, with some consensus sequences having >50–100 duplicate reads. This is likely due to the increased amplification needed to produce enough DNA from FFPE samples combined with the reduced quality of DNA extracted from FFPE samples. Here our case clearly shows that the usage of UMI or MBC can greatly increase the accuracy of detection of low-quality DNA with much improved allelic quantifications, e.g., for FFPE and ctDNA.

#### 3.1.4 Realignment, base score recalibration and estimation of sequencing coverage

Next, filtered alignments are further processed to improve the alignment quality, including local realignment around indels and base quality score recalibration using GATK. The step of local realignment is to improve the alignment quality for bases around known and suspected indel positions to reduced false positive calls. Base score recalibration is carried out to recalculate base quality scores for all sequenced reads based on known polymorphisms (e.g., SNPs from 1000G Project). The base and mapping quality scores are used to filter reads during variant calling and the fine-tuning that occurs in this step is important to ensure only high-confidence variants are called.

Base coverage information is another important parameter to assess the overall quality of TS data. Using recalibrated BAM files, one can further calculate the coverage/depth for bases within the targeted regions, using Bedtools or GATK coverage. Depending on the quality of DNA and total number of reads generated, several hundred times depth per base is often expected, although some regions may have much higher coverage or targeted rates than others. However, for ultra-deep sequencing, the depth of tens of thousands of reads is often required to detect very low frequency clones.

### 3.2 Variant calling

Once all TS pre-processing steps are completed, these high-quality alignment data are ready for variant calling. Variant calling is the process of comparing the aligned reads to a reference genome or matched normal DNA sequences to identify base pair variations. Here we describe the procedure for samples with matched normal and without matched normal separately. We then focus on variant calling parameters and filters which can be tuned accordingly to achieve the best outcome.

#### 3.2.1 A note on paired end sequencing

Whilst paired end sequencing improves the accuracy of read alignment, it can lead to overlapped regions within read pairs. This problem is especially prevalent with shorter DNA fragments. As these paired reads are from the same DNA fragment, the overlapping sections are duplicates of one another and will lead to the bases in this region being counted twice. To combat this source of error most variant callers have built in methods to handle overlaps. For example, SAMtools mpileup will set the mapping quality of one of the overlapped reads to 0 (i.e., not mapped) within this region, to ensure it is not included in the variant calling.

#### 3.2.2 Samples with matched normal

Normal refers to DNA extracted from the non-tumour tissue of the cancerous organ, however it is often that “blood derived DNA” is used as a germline control when normal tissue is unsuitable or unavailable. Five of the studies shown in Table 4 included matched tumour/normal samples [17], [22], [34], [46], [48]. This allows for the patient specific germline SNPs to be identified and disregarded in the tumour sample [57], [65]. Commonly used software for somatic variant calling includes MuTect2 [56], Strelka2 [57] and VarScan2 [58] somatic mode. These methods process normal and tumour alignment BAM files together, first by calling variants against a reference genome before determining somatic variants based on sophisticated models (e.g., mixture model), taking into consideration of factors like depth, error rate and haplotype to call high-confidence variants. However, one can always further filter produced variants using the total coverage and number of supporting reads for the sites [66], [67], [68].

Studies comparing multiple variant callers found poor overlap results between different methods [66], [67]. MuTect2 and Strelka2 seemed to perform well compared to their contemporaries, and also display a good level of concordance with one another in their SNV calls (~90%), although their indel calls were much less concordant (~55%) [68]. Thus, in some studies, variants were called using multiple callers and only these supported by at least two methods were selected [68], [69].

#### 3.2.3 Samples without matched normal

However, matched normal samples are often not available in the clinical setting, especially for retrospective FFPE or FF samples. Consequently, it is much more challenging to call reliable somatic variants in this scenario, confounded by the presence of a large number of germline SNPs. As with matched samples, variant calling starts with comparison to the reference genome identifying all possible variant positions, including germline and potential somatic calls, using VarSan2 or SAMtools [55]. Both methods can work on one or multiple sample alignments in a ‘pileup’ format generated by SAMtools, and call variants against the reference genome with filters, such as the minimum base and mapping quality, the number of supporting reads and total coverage for called sites. Another commonly used method is GATK, where the HaplotypeCaller module can be used to call variants for multiple samples effectively. HaplotypeCaller is able to call SNPs and indels simultaneously via local de-novo assembly of haplotypes in an active region. In regions with many variations detected, HaplotypeCaller reassembles the reads in that region without the use of existing mapping information. This makes the calls much more accurate, especially for different types of variants close to each other. Various variant annotation and filtering steps are then applied, outlined below, to remove low-confidence and non-significant calls, which we will expand in the following sections. Without the matched normal samples, germline variants cannot be removed as above and must be thoroughly processed to remove commonly or benign variants that are less likely to originate from the tumour. In many studies, although the matched normal samples were not available, a panel of normal DNA samples were included to help significantly remove SNPs and sequencing artefacts [15], [17], [70]. In order to increase the validity of normal samples, they should be as closely matched as possible to the study cohort e.g. sex and ethnicity.

#### 3.2.4 Calling inherited germline variants

The pipeline presented in Fig. 2 is versatile, and can also be applied for germline variant calling. However, when calling somatic variants, positions that are called in common between samples are highly likely to be SNPs/artefacts and should be removed, unless they are mutational hotspots. In contrast when calling germline variants these recurring calls should be kept if they are found within members of the same pedigree as these are likely to be inherited variants. An additional consideration with germline calls is that VAFs for these variants should be at ~50% (heterozygous, using a VAF range of 30–70% or 40–60% depending on the sequencing depth) or ~100% (homozygous). Any ‘sub-clonal’ variants should be ignored in germline variant calling. Furthermore, any genes shared across different pedigrees should be regarded as important familial gene candidates.

#### 3.2.5 Variant calling parameters and filtering

A set of important parameters need to be considered for variant calling and filtering for high-quality calls. These include,

• Number of total reads: this parameter can be used to ensure there is sufficient coverage over the position for variants to be called. Often a minimum of 20-30x depth is required for TS [71], [72], [73], [74], [75].
• Number of variant supporting reads: this parameter should be set in order to limit variants with very few supporting reads being considered. The value can be tuned based on the average coverage of the samples. Usually the minimum value ranges from 4 to 10 reads [26], [47], [76].
• Minimum base and mapping quality score: Setting a threshold for base and mapping quality scores stops poorly sequenced or aligned reads from being considered in the variant calling. The default minimum values of many programmes are set as 20–30 as these correspond to an accuracy of 99% and 99.9% respectively.
• Minimum allele frequency for called variants: Like the number of variant supporting reads, this can be used to eliminate variant positions with low levels of support. Often, a relatively low threshold (e.g., 3% with a depth of 200x) is initially used to include most of the variants, and further filtering and refinement are performed via testing a range of threshold values to choose the best cutoff value for VAF. For FFPE samples, the final threshold is set as at least 10% or even 20% across many studies [77], [78]. For FF samples this threshold can be much lower depending on overall sequencing depth [46], [50]. One should note that the tumour purity of clinical samples is often highly heterogeneous. Thus, filtering simply based on an observed VAF cutoff may not provide the most accurate way to include high-quality or exclude low-quality calls. One way to overcome this is to further adjust VAF values based on the estimates of tumour purity of clinical samples, and apply the threshold on these adjusted VAFs to filter calls for the downstream analyses. When an accurate measurement of tumour purity is not available, VAFs of mutations in many known clonal driver genes (e.g., *KRAS* and *TP53* for many solid tumours) could be used to derive a rough estimate.

Additional parameters also include:

• Strandedness of variant supporting reads: If a variant occurs within a sample, paired sequencing should show evidence of this variant on both strands. Therefore if the majority of the reads for a variant occur on only one strand (i.e., strand bias), it could suggest that variant reads are artefacts [58], [76]. In many programmes, at least one supporting read is required to be present on each strand for the called variants. In VarScan2, it is possible to require that a maximum of 90% of all reads (across reference and alternative alleles) are found on one strand, meaning positions that have a strand bias will be ignored.
• Significance score for a statistical test: Many variant callers will calculate a statistical evaluation of the likelihood of a variant differing from the reference allele [47], [76]. VarScan2 for example provides the user with a *p* value for a Fisher’s Exact Test on the observed and expected variant reads. This can be used to further eliminate low-quality calls.

These parameters can be fine-tuned based on the aims of the project and the data that is generated. Among the publications reviewed in Table 4, for example, the high depth of sequencing in Araf et al. (estimated at ~8000×) combined with error suppression allowed variants to be called with VAFs as low as 0.1% [44]. Elis et al. initially called variants of VAFs as low as 2% in matched FFPE samples, utilising the validation mode within VarScan2, followed by further customised filtering to retain high quality calls [48]. In defining the m7-FLIPI index a VAF cut-off of >10% was used. These data were generated from FFPE samples and many samples had no matched germline to filter out SNPs, meaning a robust cut-off was necessary to ensure high quality calls [17].

### 3.3 Annotation and further filtration of variants.

Following variant calling, the next step is to annotate the variants in relation to genes (e.g., within or outside a gene), codon and amino acid positions, and classify types of variants, such as nonsense, missense, exonic deletions and synonymous variants. This allows for greater understanding of their functional consequences on genes they relate to. In many TS studies, only non-silent exonic or splicing mutations are selected for further analysis, focusing on functional coding variants and mutations only. However, these criteria may vary depending on the region of interest or the purpose of the study, e.g. variants in promoter regions or UTRs of the genome.

Commonly used variant annotation methods include ANNOVAR, SNPeff, VEP and Oncotator [60], [61], [62], [79]. These methods provide rich resources of gene and regulatory annotation, functional prediction, sequence conservation and frequencies in the population level. Here we describe a general annotation and filtering workflow for variants called in a cancer TS experiment without matched normal tissue. The workflow follows as below,

1. Gene annotation: annotate variants against Ensembl or RefGene gene models, to retain all non-silent variants including those affecting splice sites and exonic indels.
2. SNP and cancer variant identification and filtering: Find variants that are overrepresented in the general population. Datasets such as dbSNP, 1000 Genome Project, NHLBI GO Exome Sequencing Project (ESP), The Genome Aggregation Database (gnomAD) [80] and ExAC [81] include the estimated frequency of variants. Any variants with minor allele frequency >1% are excluded, as these more common variants are less likely to have any oncogenic implications. Filtered variants are then annotated against the COSMIC database (a cancer mutation catalogue), allowing those variants present in dbSNP but also previously identified as cancer mutations to be retained.
3. Variant recurrence filtering: the remaining non-silent variants are still likely to contain many SNPs and sequencing artefacts. Specifically, variants that occur in many samples (e.g., >15/20% of samples) but are not known COSMIC hotspots are likely these candidates for removal. When VAFs of those variants are consistently low (e.g., <5% when UMIs are not used) across all samples, these typically represent sequencing artefacts. When recurrent variants have consistently high VAFs (over 30/40%) across all samples, this suggests that they are likely SNPs. A panel of normal samples (unmatched) sequenced alongside the tumour samples can significantly aid in reducing these recurrent variants if they also occur in the normal controls.
4. Variant and gene prioritisation: functional consequences of variants are predicted using databases such as SIFT [79], PolyPhen [80], and MutationTaster [82]. Highly scored variants are likely to have strong deleterious effects on the targeted genes, warranting further investigation. Genes with deleterious variants that are over-represented across the cohort, are potentially strongly involved in the biology of that cancer. However, care must be taken when selecting candidates for further study as confounding factors can also cause a high level of mutations in individual genes, e.g. gene length. Commonly used programmes to detect significantly mutated genes (e.g., MutSigCV and dNdScv) can still be applied to TS data to prioritise candidate genes.

### 3.4 Estimation of background error rate

The sensitivity of NGS is in the regions of VAF 1% [83], [84]. However, there is a need in some studies to identify variants with much lower VAFs, e.g., to detect very small subclonal and minimal residual disease (MRD) mutations. To achieve this, higher depth of sequencing is usually required, and a comprehensive strategy is needed to differentiate between genuine calls and background sequencing artefacts or the background noise rate at VAFs < 1%. Tawana et al. applied ultra-deep TS (depth of 10,000–100,000×) to investigate pre-existing leukaemic clones and disease evolution in sequential acute myeloid leukaemia biopsies [16]. Two independent strategies were used to account for the noise level: first, the reference and variant allele supporting reads of targeted variants were compared among sequential samples and also with a panel of non-related DNA, to ensure these were not recurrent sequencing artefacts; second, the reads for the variants of interest were also compared with those of variants detected within surrounding base pairs, to exclude false positive calls due to background noise, with the background noise rate also calculated at around 0.20%. This successfully led to the discovery of a small clone (3% of cells) harbouring a *TET2* nonsense mutation, which expanded and became the dominant clone at a later stage.

There are also software packages available that try to control for background mutation rate in non-matched samples using a panel of normals [85], [86]. Integrated digital error suppression (iDES) is one such method that utilises a combination of CAPP-Seq molecular barcodes and background ‘polishing’ that is able to reduce the error rate further than either method used in isolation [86]. The molecular barcodes allowed an *in silico* reassembly of the original DNA duplex reducing sequencing artefacts, whilst the polishing was carried out using a novel method, which removed variants that were statistically indistinguishable from background levels found in a panel of normals. Whilst combining the methods resulted in the best improvement in background error rate reduction (~15 fold) the polishing alone was shown to improve the error by ~3 fold, similar to the effect of the molecular barcodes. Therefore, the iDES polishing alone could be easily included in existing variant calling pipelines to reduce the error rate. The iDES software can be found at: http://cappseq.stanford.edu/ides/.

## 4 Summary and outlook

TS is a powerful and invaluable tool for mutational detection, and it has been widely applied in cancer research and clinical studies across many cancer types. Compared to its counterparts WGS and WES, TS can screen a large number of samples at much reduced costs and computational burden. This makes it extremely attractive for clinical research with fast turnaround. Until the WGS/WES cost drops to an affordable rate for large-scale applications, TS will continue to be the main genomic tool in disease genotyping. The capability of TS to detect subclonal mutations, sequencing ctDNA and for minimal residual disease monitoring also makes it a useful genetic tool to track disease evolution and study drug resistance.

However, the use of TS in routine clinical practice is still in its infancy. Whilst these data demonstrate TS can generate clinically relevant results, the key question remains whether TS can be used as a stand-alone genomic diagnostic tool. We believe that this depends on the clinical questions under investigation. When clonal mutations are explored for diagnosis and targeted therapies, TS is accurate with a normal depth of 300-500x. When subclonal events and/or MRD serve as the focus, we recommend that TS should be validated and interpreted with other approaches (e.g., digital PCR). As shown above rarer events can be observed with very high depth sequencing, but the levels of sequencing artefacts will also increase. In these cases, the estimation of the background noise level is crucial in determining an appropriate cutoff for acceptable variants. However, for FFPE samples, we argue that a cutoff of VAF value of 10% should be implemented in cases where these samples are investigated for diagnosis and prognosis due to the poor DNA quality of these samples. Note that tumour purity should also be accounted for if necessary.

One should also be aware of the limitations of TS. Due to its nature, targeting pre-defined genes, it is less useful or efficient for the detection of large-scale rearrangements (e.g., structural variants) and copy number changes, compared to the whole-genome profiling (e.g., WGS/WES). However, for known common translocation events (e.g., t(14;18) in follicular lymphoma), one can still design primers or probes to capture regions spanning the breakpoints, and TS should be able to detect these events by identifying reads that cleanly span the breakpoints. For copy number changes, normalised sequencing depth of coverage can still be used to infer copy number status, using software such as CONTRA [87] and SeqCNV [88]. However, this still remains challenging, strongly determined by the TS quality and uniformity of coverage across genes. Although not suitable for the detection of novel genomic events, TS remains as a powerful and economical tool to identify known events in patients.

The current challenge and bottleneck for large-scale cross-centre TS applications is the lack of gold-standard methods for identifying cancer-associated mutations. Individual laboratories tend to develop their own pipelines with different parameters used, often leading to a poor level of overlapped results. Thus, there is an urgent need for reliable and standard data processing and mining methods that can bring TS into routine clinical practice. We argue that benchmarking studies are urgently needed to address this issue. Initiatives such as the ICGC-TCGA DREAM Genomic Mutation Calling Challenge on WGS data are first steps in this direction [89]. Once we have the standard off-the-shelf TS analysis methods and pipelines accepted by the community, these can then be widely used across many research and clinical settings.

## Authors contributions

F.B-C and J.W. conceived and designed the study. F.B.C performed the study and collated the data. F.B.C and J.W. wrote the manuscript. All authors contributed to the revision of the manuscript.

## Declaration of Competing Interest

The authors declare that they have no known competing financial interests or personal relationships that could have appeared to influence the work reported in this paper.
